# Epidemiology and outcome of rib fractures: a nationwide study in the Netherlands

**DOI:** 10.1007/s00068-020-01412-2

**Published:** 2020-06-06

**Authors:** Jesse Peek, Reinier B. Beks, Falco Hietbrink, Mirjam B. De Jong, Marilyn Heng, Frank J. P. Beeres, Frank F. A. IJpma, Loek P. H. Leenen, Rolf H. H. Groenwold, Roderick M. Houwert

**Affiliations:** 1 Department of Surgery, University Medical Center Utrecht, PO Box 85500, 3508 GA Utrecht, The Netherlands; 2 Department of Orthopaedic Surgery, Harvard Medical School Orthopedic Trauma Initiative, Massachusetts General Hospital, Boston, MA USA; 3 Department of Orthopedic and Trauma Surgery, Cantonal Hospital Lucerne, Lucerne, Switzerland; 4 Department of Trauma Surgery, University Medical Center Groningen, Groningen, The Netherlands; 5 Department of Clinical Epidemiology, Leiden University Medical Center, Leiden, The Netherlands

**Keywords:** Rib fractures, Epidemiology, Incidence, Mortality, Outcome

## Abstract

**Purpose:**

Rib fractures following thoracic trauma are frequently encountered injuries and associated with a significant morbidity and mortality. The aim of this study was to provide current data on the epidemiology, in-hospital outcomes and 30-day mortality of rib fractures, and to evaluate these results for different subgroups.

**Methods:**

A nationwide retrospective cohort study was performed with the use of the Dutch Trauma Registry which covers 99% of the acutely admitted Dutch trauma population. All patients aged 18 years and older admitted to the hospital between January 2015 and December 2017 with one or more rib fractures were included. Incidence rates were calculated using demographic data from the Dutch Population Register. Subgroup analyses were performed for flail chest, polytrauma, primary thoracic trauma, and elderly patients.

**Results:**

A total of 14,850 patients were admitted between 2015 and 2017 with one or more rib fractures, which was 6.0% of all trauma patients. Of these, 573 (3.9%) patients had a flail chest, 4438 (29.9%) were polytrauma patients, 9273 (63.4%) were patients with primary thoracic trauma, and 6663 (44.9%) were elderly patients. The incidence rate of patients with rib fractures for the entire cohort was 29 per 100.000 person-years. The overall 30-day mortality was 6.9% (*n* = 1208) with higher rates observed in flail chest (11.9%), polytrauma (14.8%), and elderly patients (11.7%). The median hospital length of stay was 6 days (IQR, 3–11) and 37.3% were admitted to the intensive care unit (ICU).

**Conclusions:**

Rib fractures are a relevant and frequently occurring problem among the trauma population. Subgroup analyses showed that there is a substantial heterogeneity among patients with rib fractures with considerable differences regarding the epidemiology, in-hospital outcomes, and 30-day mortality.

## Introduction

Thoracic injuries are the third most common injuries in trauma patients after head and extremity injuries [1]. Rib fractures are considered the most prevalent injury following thoracic trauma and can occur in a broad spectrum of severity, ranging from a single isolated fracture to flail chest. Fractured ribs are associated with a significant morbidity and even isolated fractures can result in severe pain and long-term disability [2, 3]. The mortality rates among hospitalized patients with rib fractures range from 10 to 22%, with higher mortality rates among the elderly and in patients with flail chest [4–8].

Over the past decades, there has been increasing interest concerning different treatment strategies for rib fractures with a large number of studies reporting on the surgical treatment of these injuries [9, 10]. However, little is known about the current incidence of rib fractures and how extensive the problem is among the trauma population. Existing studies on the epidemiology of rib fractures do not report on absolute incidence rates and are mainly from the beginning of this millennium.

Previous studies have shown worse outcomes in the elderly with rib fractures and patients with a flail chest [5, 11]. Furthermore, differences in outcome are to be expected in polytrauma patients with rib fractures as compared to monotrauma patients. Consequently, there is a large heterogeneity present among patients with rib fractures, which requires reporting on specific subgroups. However, in the current literature there is insufficient data available regarding incidence rates and differences in outcome between subgroups of patients with rib fractures.

Therefore, the aim of this nationwide study was to provide current data on the incidence rates and outcomes of rib fractures and to compare these results for the different subgroups: flail chest, polytrauma, primary thoracic trauma, and elderly patients.

## Methods

The Medical Ethical Review Board of the University Medical Center Utrecht approved this study and granted a waiver of consent (METC number WAG/dgv/18/019105). This article was written in adherence to the STROBE (Strengthening the Reporting of Observational Studies in Epidemiology) statement [12].

A nationwide retrospective cohort study was performed with the use of the Dutch Trauma Registry (DTR). The DTR was founded in 2007 and is maintained by the Dutch Trauma Network of Acute Care with the general purpose of monitoring trauma care with a standardized registry and to ensure high quality care for severely injured patients. The DTR covers approximately 99% of all hospitals in the Netherlands and prospectively collects data on all trauma patients who are admitted to the hospital after presenting to the emergency department, within 48 h after trauma. Patients presented to the emergency department by pre-hospital Emergency Medical Services, as well as by self-admission, are included in the DTR. Excluded are patients declared dead on arrival, who are discharged home, and those admitted to the hospital for reasons other than their traumatic injury [13]. To determine the incidence rate of rib fractures requiring hospital admission, national demographic data were obtained using the Dutch Population Register from the Central Bureau of Statistics [14].

All patients aged 18 years and older admitted to the hospital between January 2015 and December 2017 with one or more rib fractures were identified using Abbreviated Injury Scale (AIS) codes for rib fractures. Eligible patients were divided into four groups: flail chest, polytrauma, primary thoracic trauma, and elderly. Flail chest was defined as three or more sequential rib fractures in at least two places. Polytrauma was defined as an Injury Severity Score (ISS) of 16 or higher. Primary thoracic trauma was defined as an AIS thorax score higher than the AIS score of all other domains. Elderly patients were defined as patients aged 65 years and older.

The following baseline variables were obtained from the DTR: age at trauma, sex, American Society of Anesthesiologists (ASA) score, mechanism of injury, mode of transport (i.e., ambulance, own transport, or trauma helicopter) and involvement of the Mobile Medical Team (MMT), Glasgow Coma Scale (GCS), vital parameters upon time of admission (i.e., systolic blood pressure and respiratory rate), need for emergency intervention, fracture and injury-related characteristics including number of fractured ribs and presence of a flail chest, ISS, AIS scores for all body regions, and Revised Trauma Score (RTS). In the Netherlands, the MMT consists of a trauma surgeon or anesthesiologist and a trained nurse to provide acute care on the site of the accident. The Revised Trauma Score is a widely used 13-point scoring tool to determine the initial trauma severity based on the GCS, systolic blood pressure, and respiratory rate. A lower score reflects a higher severity of injury.

The in-hospital outcome variables obtained were hospital length of stay (HLOS), admission to intensive care unit (ICU), ICU length of stay (ILOS), mortality, and Glasgow Outcome Scale (GOS) score at the time of hospital discharge.

Data were analyzed using descriptive statistics and presented as frequencies with percentages for categorical data, means with standard deviations (SD) for normally distributed continuous data, and medians with interquartile ranges (IQR) for non-normally distributed continuous data. The Shapiro–Wilk test and quantile–quantile plots were applied to detect deviations from the normal distribution. The incidence rate was calculated by dividing the total number of patients with rib fractures by the total Dutch population during the study period. Incidence rates were expressed per 100,000 person-years. Statistical analyses were performed using SPSS statistical software (SPSS 23.0; IBM Inc., Armonk, NY, USA).

## Results

Between January 2015 and December 2017, a total of 245,548 patients were acutely admitted to the hospital through the emergency department after suffering from trauma. Of these, 14,850 patients had rib fractures (6.0%). There were 573 (3.9%) patients with a flail chest, 4438 (29.9%) with polytrauma, 9273 (62.4%) with primary thoracic trauma, and 6663 (44.9%) were elderly patients.

The incidence rate of rib fractures requiring hospital admission for the entire cohort was 29 per 100,000 person-years. The median age at the time of trauma was 62 (IQR 49–75) years and 67.8% (*n* = 10,073) were male. The overall 30-day mortality was 6.9% (*n* = 1028). The baseline characteristics and outcomes are presented in Tables 1, 4, respectively.

**Table 1:** Demographic and pre-hospital data of patients with rib fractures stratified by subgroups

Variable	Total patients	Flail chest		Polytrauma (ISS ≥ 16)		Primary thoracic trauma		Elderly (≥ 65 years)	
		Yes	No	Yes	No	Yes	No	Yes	No
	*n* = 14,850	*n* = 573	*n* = 14,277	*n* = 4438	*n* = 10,412	*n* = 9273	*n* = 5577	*n* = 6663	*n* = 8187
Demographic data									
Age at trauma, median (IQR)	62 (49–75)	62 (51–73)	62 (49–75)	59 (46–73)	63 (51–76)	62 (51–75)	61 (47–75)	77 (70–84)	51 (42–57)
Sex, *n* (%)									
Male	10,073 (67.8)	403 (70.2)	9.670 (67.7)	3131 (70.5)	6942 (66.6)	6379 (68.8)	3,694 (66.2)	3,819 (57.3)	6254 (76.4)
Female	4777 (32.2)	171 (29.8)	4.606 (32.3)	1307 (29.5)	3470 (33.3)	2894 (31.2)	1,883 (33.8)	2,844 (42.7)	1933 (23.6)
Comorbidity ASA, *n* (%)^*^									
Normal healthy patient	5027 (33.9)	190 (33.2)	4837 (33.9)	1649 (37.2)	3378 (32.4)	3069 (33.1)	1,958 (35.1)	882 (13.2)	4145 (50.6)
Mild systemic disease	5518 (37.2)	189 (33.0)	5329 (37.3)	1527 (34.4)	3991 (38.3)	3464 (37.4)	2,054 (36.8)	3,329 (50.0)	2189 (26.7)
Moderate systemic disease	1569 (10.6)	49 (8.6)	1520 (10.6)	429 (9.7)	1140 (10.9)	981 (10.6)	588 (10.5)	1,193 (17.9)	376 (4.6)
Severe systemic disease	131 (0.9)	6 (1.0)	125 (0.9)	36 (0.8)	95 (0.9)	76 (0.8)	55 (1.0)	115 (1.7)	16 (0.2)
Moribund patients	5 (0)	0 (0)	5 (0)	1 (0)	4 (0)	4 (0)	1 (0)	4 (0.1)	1 (0)
Pre-hospital data									
Glasgow Coma Scale, median (IQR)	15 (15–15)	15 (14–15)	15 (15–15)	15 (12–15)	15 (15–15)	15 (15–15)	15 (14–15)	15 (15–15)	15 (15–15)
Mode of transport, *n* (%)^*^									
Ambulance	10,471 (70.5)	414 (72.3)	10,057 (70.4)	3244 (73.1)	7227 (69.4)	6351 (68.5)	4,120 (73.9)	4,848 (72.8)	5623 (68.7)
Own transport	2228 (15.0)	58 (10.1)	2170 (15.2)	185 (4.2)	2043 (4.2)	1759 (19.0)	469 (8.4)	947 (14.2)	1281 (15.6)
Trauma helicopter	166 (1.1)	13 (2.3)	153 (1.1)	137 (3.1)	29 (0.3)	52 (0.6)	114 (2.0)	51 (0.8)	115 (1.4)
Ambulance with helicopter MMT	862 (5.8)	60 (10.5)	802 (5.6)	690 (15.5)	172 (1.7)	305 (3.3)	557 (10.0)	259 (3.9)	603 (7.4)
Other	48 (0.3)	3 (0.5)	45 (0.3)	10 (0.2)	38 (0.4)	35 (0.4)	13 (0.2)	21 (0.3)	27 (0.3)
Involvement of MMT, *n* (%)	1431 (9.6)	477 (83.2)	13,058 (91.5)	1129 (25.4)	302 (2.9)	505 (5.4)	926 (16.6)	408 (6.1)	1023 (12.5)
Intubation on scene, *n* (%)	712 (4.8)	70 (12.2)	642 (4.5)	655 (14.8)	57 (0.5)	171 (1.8)	541 (9.7)	223 (3.3)	489 (6.0)
Emergency intervention, *n* (%)									
Thoracotomy/laparotomy	144 (1.0)	11 (1.9)	133 (0.9)	136 (3.1)	8 (0.1)	47 (0.5)	97 (1.7)	21 (0.3)	123 (1.5)
Craniotomy	99 (0.7)	8 (1.4)	91 (0.6)	99 (2.2)	0 (0)	0 (0)	99 (1.8)	35 (0.5)	64 (0.8)
Other	548 (3.7)	46 (8.0)	502 (3.5)	448 (10.1)	100 (1.0)	226 (2.4)	322 (5.8)	163 (2.4)	385 (4.7)

*ASA* American Society of Anesthesiologists, *IQR* interquartile range, *ISS* Injury Severity Score, *MMT* mobile medical team

^*^ Percentages may not add up to 100 due to missing data

### Flail chest

The incidence rate of patients with flail chest was 1 per 100,000 person-years. Among the 573 patients with a flail chest, the median age was 62 (IQR 51–73) years and 70.2% (*n* = 402) were male. The median ISS was 17 (IQR 10–27) with a median AIS thorax of 3 (IQR 3–3) (Table 2). The most common mechanisms of injury were low energy falls (22.5%, *n* = 129), followed by bicycle accidents (14.3%, *n* = 82), high energy falls (13.6%, *n* = 78), and motor vehicle accidents (10.8%, *n* = 62) (Table 3). Among flail chest patients, the median HLOS was 9 (IQR 5–16) days and 63.5% required admission to the ICU with a median ILOS of 3 (IQR 2–6) days. The 30-day mortality was 11.9% (*n* = 68).

**Table 2:** Fracture- and injury-related characteristics of patients with rib fractures stratified by subgroups

**Variable**	Total patients	Flail chest		Polytrauma (ISS ≥ 16)		Primary thoracic trauma		Elderly (≥ 65 years)	
		Yes	No	Yes	No	Yes	No	Yes	No
	*n* = 14,850	*n* = 573	*n* = 14,277	*n* = 4438	*n* = 10,412	*n* = 9273	*n* = 5577	*n* = 6663	*n* = 8187
Number of fractured ribs, *n* (%)									
1	2662 (17.9)	N/A	2654 (18.6)	371 (8.4)	2291 (22.0)	888 (9.6)	1774 (31.8)	1153 (17.3)	1509 (18.4)
2	2627 (17.7)	N/A	2514 (18.3)	430 (9.7)	2197 (21.1)	1374 (14.8)	1253 (22.5)	1209 (18.1)	1418 (17.3)
≥ 3	9561 (64.4)	573 (100)	9009 (63.1)	4438 (81.9)	5924 (56.8)	7011 (75.6)	2550 (45.7)	4301 (64.6)	5260 (64.3)
Flail chest, *n* (%)									
Yes	573 (3.9)	573 (100)	0 (0)	312 (7.0)	261 (2.5)	416 (4.5)	157 (2.8)	259 (3.9)	314 (3.8)
No	14,277 (96.1)	0 (0)	14,277 (100)	4261 (93.0)	10,151 (97.5)	8857 (95.5)	5420 (97.2)	6404 (96.1)	7873 (96.2)
ISS, median (IQR)	12 (9–17)	17 (10–27)	11 (9–17)	22 (17–29)	9 (8–13)	10 (9–14)	18 (9–27)	10 (9–17)	13 (9–18)
Polytrauma (ISS ≥ 16), *n* (%)									
Yes	4438 (29.9)	312 (54.5)	4216 (28.9)	4438 (100)	0 (0)	1642 (17.7)	2796 (50.1)	1787 (26.8)	2651 (32.4)
No	10,412 (70.1)	261 (45.5)	10,151 (71.1)	0 (0)	10,412 (100)	7631 (82.3)	2781 (49.9)	4876 (73.2)	5536 (67.6)
AIS, median (IQR)									
Head	0 (0–1)	0 (0–2)	0 (0–1)	1 (0–3)	0 (0–0)	0 (0–0)	1 (1–3)	0 (0–1)	0 (0–1)
Face	0 (0–0)	0 (0–0)	0 (0–0)	0 (0–1)	0 (0–0)	0 (0–0)	0 (0–1)	0 (0–0)	0 (0–0)
Thorax	3 (2–3)	3 (3–3)	3 (2–3)	3 (3–4)	3 (2–3)	3 (2–3)	2 (2–3)	3 (2–3)	3 (2–3)
Abdomen	0 (0–0)	0 (0–0)	0 (0–0)	0 (0–0)	0 (0–0)	0 (0–0)	0 (0–0)	0 (0–0)	0 (0–0)
Spine	0 (0–0)	0 (0–2)	0 (0–0)	0 (0–2)	0 (0–0)	0 (0–0)	0 (0–2)	0 (0–0)	0 (0–0)
Extremities	0 (0–0)	0 (0–1)	0 (0–0)	0 (0–2)	0 (0–0)	0 (0–0)	0 (0–2)	0 (0–0)	0 (0–0)

*AIS* Abbreviated Injury Scale, *IQR* interquartile range, *ISS* Injury Severity Score, *N/A* not applicable

**Table 3:** Mechanism of injury in patients with rib fractures stratified by subgroups

Variable	Total patients *n* = 14,850	Flail chest		Polytrauma (ISS ≥ 16)		Primary thoracic trauma		Elderly (≥ 65 years)	
		Yes	No	Yes	No	Yes	No	Yes	No
		*n* = 573	*n* = 14,277	*n* = 4438	*n* = 10,412	*n* = 9273	*n* = 5577	*n* = 6663	*n* = 8187
Mechanism of injury, *n* (%)									
Motor vehicle accident	1456 (9.8)	62 (10.8)	1,394 (9.8)	696 (15.7)	760 (7.3)	752 (8.1)	704 (12.6)	457 (6.9)	999 (12.2)
Motorcycle accident	589 (4.0)	34 (5.9)	555 (3.9)	225 (5.1)	364 (3.5)	354 (3.8)	235 (4.2)	54 (0.8)	535 (6.5)
Bicycle accident	2327 (15.7)	82 (14.3)	2245 (15.7)	713 (16.1)	1614 (15.5)	1373 (14.8)	954 (17.1)	1042 (15.6)	1285 (15.7)
Low energy fall	4474 (30.1)	129 (22.5)	4345 (30.4)	725 (16.3)	3749 (36.0)	3155 (34.0)	1319 (23.7)	2850 (42.8)	1624 (19.8)
High energy fall	1938 (13.1)	78 (13.6)	1860 (13.0)	850 (19.2)	1088 (10.4)	1017 (11.0)	921 (16.5)	759 (11.4)	1179 (14.4)
Other	1517 (10.2)	58 (10.1)	1459 (10.2)	539 (12.1)	978 (9.4)	898 (9.7)	619 (11.1)	438 (6.6)	1079 (13.2)

Percentages may not add up to 100 due to missing data

*ISS* Injury Severity Score, *n*, number

### Polytrauma

The incidence rate of polytrauma patients with rib fractures was 9 per 100,000 person-years. Among the 4438 polytrauma patients, the median age was 59 (IQR 46–73) years and 70.5% (*n* = 3131) were male. The median ISS was 22 (IQR 17–29) with a median AIS thorax of 3 (IQR 3–4) (Table 2). The most common mechanisms of injury were high energy falls (19.2%, *n* = 850), followed by low energy falls (16.3%, *n* = 725), bicycle accidents (16.1%, *n* = 713), and motor vehicle accidents (15.7%, *n* = 696) (Table 3). Among polytrauma patients, the median HLOS was 10 (IQR 5–18) days and 65.8% (*n* = 2918) required admission to the ICU with a median ILOS of 3 (IQR 2–9) days. The 30-day mortality was 14.8% (*n* = 655).

### Primary thoracic trauma

The incidence rate of primary thoracic trauma patients with rib fractures was 18 per 100,00 person-years. Among the 9273 patients with primary thoracic trauma, the median age was 62 (IQR 51–75) years and 68.8% (*n* = 6378) were male. The median ISS was 10 (IQR 9–14) with a median AIS thorax of 3 (IQR 2–3) (Table 2). The most common mechanisms of injury were low energy falls (34.0%, *n* = 3155), followed by bicycle accidents (14.8%, *n* = 1373), high energy falls (11.0%, *n* = 1017), and motor vehicle accidents (8.1%, *n* = 752) (Table 3). Among patients with primary thoracic trauma, the median HLOS was 6 (IQR 3–10) days and 28.7% required admission to the ICU with a median ILOS of 2 (IQR 2–4) days. The overall 30-day mortality was 4.2% (*n* = 393).

### Elderly

The incidence rate of elderly patients with rib fractures was 72 per 100,000 person-years. Among the 6,663 elderly patients, the median age was 77 (IQR 70–84) years and 57.3% (*n* = 3819) were male. The median ISS was 10 (9–17) with a median AIS thorax of 3 (IQR 2–3) (Table 2). The most common mechanisms of injury were low energy falls (42.8%, *n* = 2850), followed by bicycle accidents (15.6%, *n* = 1042), high energy falls (11.4%, *n* = 759), and motor vehicle accidents (6.9%, *n* = 457) (Table 3). Among elderly patients, the median HLOS was 7 (IQR 4–12) days and 30.0% required admission to the ICU (*n* = 2001) with a median ILOS of 3 (IQR 2–6) days. The 30-day mortality was 11.7% (*n* = 782) (Table 4).

**Table 4:** In-hospital outcomes and 30-day mortality of patients with rib fractures stratified by subgroups

Variable	Total patients *n* = 14,850	Flail chest		Polytrauma (ISS ≥ 16)		Primary thoracic trauma		Elderly (≥ 65 years)	
		Yes	No	Yes	No	Yes	No	Yes	No
		*n* = 573	*n* = 14,277	*n* = 4438	*n* = 10,412	*n* = 9273	*n* = 5577	*n* = 6663	*n* = 8187
Admission to trauma center, *n* (%)	5533 (37.3)	304 (53.1)	5229 (36.6)	3022 (68.1)	2511 (24.1)	2638 (28.4)	2895 (51.9)	2044 (30.7)	3489 (42.6)
Admission to ICU, *n* (%)	4854 (32.7)	363 (63.4)	4491 (31.5)	2918 (65.8)	1936 (18.6)	2658 (28.7)	2196 (39.4)	2001 (30.0)	2853 (34.8)
HLOS, median (IQR)	6 (3–11)	9 (5–16)	6 (3–11)	10 (5–18)	5 (3–9)	6 (3–10)	7 (3–13)	7 (4–12)	5 (3–10)
ILOS, median (IQR)	3 (2–6)	3 (2–6)	3 (2–6)	3 (2–9)	2 (2–3)	2 (2–4)	3 (2–9)	3 (2–6)	3 (2–6)
Mortality (30-day), *n* (%)	1028 (6.9)	68 (11.9)	960 (6.7)	655 (14.8)	373 (3.6)	393 (4.2)	635 (11.4)	782 (11.7)	246 (3.0)
Destination after discharge, *n* (%)									
Home	9781 (65.9)	276 (48.2)	9505 (66.6)	1902 (42.9)	7879 (75.7)	6820 (73.5)	2961 (53.1)	3544 (53.2)	6237 (76.2)
Nursing home	1210 (8.2)	45 (7.9)	1165 (8.2)	407 (9.2)	803 (7.7)	654 (7.1)	454 (9.9)	1044 (15.87)	166 (2.1)
Rehabilitation clinic	976 (6.6)	44 (7.7)	841 (5.9)	527 (11.9)	358 (3.4)	338 (3.6)	547 (9.8)	491 (7.4)	394 (4.8)
Other hospital	134 (0.9)	12 (2.1)	122 (0.9)	93 (2.1)	41 (0.4)	93 (1.0)	77 (1.4)	60 (0.9)	74 (0.9)
GOS Score, *n* (%)									
Good recovery	4198 (28.3)	113 (19.7)	4085 (28.6)	727 (16.4)	3471 (33.3)	2984 (32.2)	1214 (21.8)	1648 (24.7)	2550 (31.1)
Moderate disability	5942 (40.0)	220 (38.4)	5722 (40.1)	1733 (39.0)	4209 (40.4)	3703 (39.9)	2239 (40.1)	2667 (40.0)	3275 (40.0)
Severe disability	832 (5.6)	57 (9.9)	775 (5.4)	623 (14.0)	209 (2.0)	284 (3.1)	548 (9.8)	338 (5.1)	494 (6.0)
Persistent vegetative state	21 (0.1)	0 (0)	21 (0.1)	18 (0.4)	3 (0.0)	3 (0.0)	18 (0.3)	8 (0.1)	13 (0.2)
Death	749 (5.0)	48 (8.4)	701 (4.9)	550 (12.4)	199 (1.9)	245 (2.6)	504 (9.0)	533 (8.0)	216 (2.6)

Percentages may not add up to 100 due to missing data

*GOS* Glasgow Outcome Scale, *HLOS* hospital length of stay, *ICU* intensive care unit, *IQR* interquartile range, *ILOS* intensive care unit length of stay

## Discussion

This nationwide study shows that rib fractures occur in a very heterogeneous patient population. Rib fractures should be regarded as a marker of severe injury as 30% of the patients sustained multiple injuries. Furthermore, this study shows that rib fractures impose a severe burden on society, as 45% were elderly patients with an incidence rate of 72 per 100,00 person-years and a mean hospital length of stay of 7 days. Although flail chest was present in only 3.9% of the patients, it should be considered as a different entity due to the high mortality rate and prolonged hospital length of stay.

Previous studies on the epidemiology of rib fractures are mainly from the beginning of this millennium. Although absolute population-based incidence rates are lacking, these studies described that rib fractures are identified in approximately 10%–40% of all trauma patients [7, 15, 16]. With the present study, we demonstrate that 6.0% of all admitted trauma patients sustained fractured ribs following thoracic trauma. Although non-admitted patients with rib fractures were not included, it is likely that the current incidence (29 per 100,000 person-years) is lower than that previously described. In addition, this is the first study reporting on the exact incidence rate of flail chest in patients with rib fractures.

In the current literature, the reported mortality of patients with rib fractures requiring hospital admission ranges between 10 and 22%, with higher rates observed in the elderly patients and patients with a flail chest [5, 7, 16]. This nationwide study demonstrates an overall 30-day mortality of 6.9%, which is lower than previously reported mortality rates [16]. The decrease in mortality is thought to be a consequence of implementation of trauma systems and the extensively improved trauma and critical care resulting in survival of previously lethal injuries [17–20]. Furthermore, since patients not admitted to hospital were not included in this analysis, the overall mortality risk of rib fractures among the general population is expected to be even lower.

The findings of our subgroup analyses illustrate the considerable clinical heterogeneity among patients with rib fractures and emphasizes the importance of subgroup identification. Flail chest patients had a higher mortality rate compared to patients without a flail chest (11.9% vs. 6.7%). However, half of the patients with a flail chest were considered polytrauma which could in part account for the higher mortality. Greater differences might be demonstrated when distinguishing between a radiological and clinical flail chest. However, this distinction is not made in the DTR. Still, the results showed substantial differences between patients with and without a flail chest regarding total HLOS (median, 9 vs. 6 days) and need for intensive care admission (53.1% vs. 36.6%). Polytrauma patients tend to be younger compared to non-polytrauma patients and have an almost threefold risk of suffering a flail chest, indicating both more severe extra-thoracic and thoracic injury. Patients with primary thoracic trauma appear to be younger and have a lower mortality rate than those with extra-thoracic injuries (4.2% vs. 11.4%), which emphasizes the impact of the extra-thoracic injury on the outcome.

Elderly patients had a lower median ISS compared to their younger counterparts (10 vs. 13) and only a third were polytrauma patients. Nevertheless, the elderly showed to have a considerably higher mortality rate (11.7% vs. 3.0%) as well as a longer HLOS than patients under 65 years of age (7 vs. 5 days). This illustrates the high clinical impact of rib fractures on the elderly population, and once again emphasizes the importance of subgroup identification. Furthermore, with the increase in aging population, rib fractures might impose the largest burden of disease after hip fractures in the elderly trauma population.

This study has several limitations. First, data from registries are subject to miscoding and incomplete data. However, a recent study of Olthof et al. reported that the reliability of the registered AIS codes in the DTR was ‘substantial’ (intraclass correlation coefficient (ICC) = 0.70), and ‘almost perfect’ for the registered ISS (ICC = 0.84) and survival status (Cohen’s *κ* = 0.82) [21]. Second, the total incidence of rib fractures might be underestimated, as the DTR only registers patients who have been admitted to a hospital, leaving out patients with rib fractures without the need for admission. Nevertheless, with the present study, we provide data on the absolute incidence rate of trauma patients admitted with rib fractures, as 99% of all Dutch hospitals are affiliated with the DTR. Third, since this study represented hospitalized patients only, caution should be exercised when comparing the results with other studies, as the incidence rates depends on the design of health-care systems, selection of patients, and inclusion criteria of the different trauma registries. Fourth, data on complications and information about the cause of death cannot be extracted from the DTR. Fifth, as the DTR does not record the interventions performed during hospital admission, we were not able to determine the incidence and outcomes of patients who received rib fixation.

This epidemiological study reports on the population-based incidence rates of rib fractures and demonstrates that rib fractures still remain a frequently occurring injury associated with a significant morbidity. By the stratification of our subgroups, we have shown that there is still substantial mortality among flail chest, polytrauma, and elderly patients, while patients with primary thoracic trauma have lower mortality rates. Furthermore, as the general population continues to increase in age, it is to be expected that more elderly patients with fractured ribs require clinical care. The average hospital stay is still 6 days and more than one-third of all patients require intensive care treatment. These findings indicate that rib fractures are a relevant and frequently occurring problem among the trauma population.

## Data Availability

The data used to support the findings of this study are restricted by the Dutch Trauma Registry (DTR) maintained by the Dutch Trauma Network of Acute Care. Data are available from the Dutch Trauma Registry for researchers who meet the criteria for access to confidential data. Requests for access to these data should be made to DTR.
